# Correction: Zhang et al. Experimental and Numerical Studies on Hot Compressive Deformation Behavior of a Cu–Ni–Sn–Mn–Zn Alloy. *Materials* 2023, *16*, 1445

**DOI:** 10.3390/ma16247555

**Published:** 2023-12-08

**Authors:** Yufang Zhang, Zhu Xiao, Xiangpeng Meng, Lairong Xiao, Yongjun Pei, Xueping Gan

**Affiliations:** 1 State Key Laboratory for Powder Metallurgy, Central South University, Changsha 410083, China; 2 School of Materials Science and Engineering, Central South University, Changsha 410083, China; 3 Key Laboratory of Non-Ferrous Metal Materials Science and Engineering, Ministry of Education, Changsha 410083, China; 4 Ningbo Boway Alloy Material Co., Ltd., Ningbo 315135, China

In the original publication [1], there was a mistake in Figure 5 as published. We found that we had accidentally duplicated plot a as plot c in the process of grouping the fitted curves. The corrected Figure 5 appears below. However, we believe that this does not significantly change the scope or message of the paper. The authors apologize for any inconvenience caused and state that the scientific conclusions are unaffected. This correction was approved by the Academic Editor. The original publication has also been updated.

## Figures and Tables

**Figure 5:**
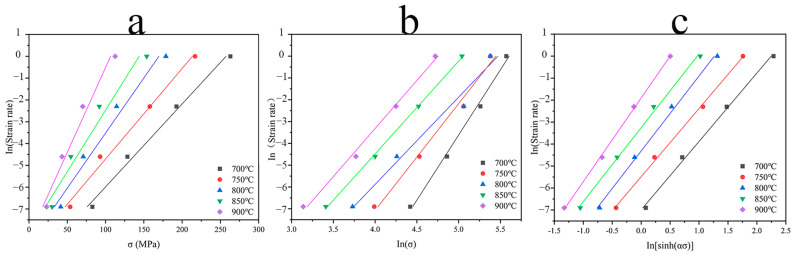
Relationship between peak stress and strain rate: (**a**) $\;\ln\dot{\varepsilon }\sim \sigma_{p}$ curve; (**b**) $\ln\dot{\varepsilon }\sim \ln\sigma_{p}$ curve; (**c**) $\;\ln\dot{\varepsilon }\sim \ln[\sinh(\alpha \sigma_{p})]$ curve.
